# Retrospective 1- to 8-Year Follow-Up Study of Complete Oral Rehabilitation Using Monolithic Zirconia Restorations with Increased Vertical Dimension of Occlusion in Patients with Bruxism

**DOI:** 10.3390/jcm11185314

**Published:** 2022-09-09

**Authors:** Shlomo Matalon, Hadas Heller, Ilan Beitlitum, Evgeny Weinberg, Alona Emodi-Perlman, Shifra Levartovsky

**Affiliations:** 1 Department of Oral Rehabilitation, The Maurice and Gabriela Goldschleger School of Dental Medicine, Sackler Faculty of Medicine, Tel Aviv University, Tel Aviv 6997801, Israel; 2 Department of Periodontology and Dental Implantology, Maurice and Gabriela Goldschleger School of Dental Medicine, Tel Aviv University, Tel Aviv 6997801, Israel; 3 Department of Oral Biology, The Maurice and Gabriela Goldschleger School of Dental Medicine, Sackler Faculty of Medicine, Tel Aviv University, Tel Aviv 6997801, Israel

**Keywords:** oral rehabilitation, veneered zirconia, non-veneered zirconia, bruxism, vertical dimension of occlusion

## Abstract

Aim: The aim of this paper is to perform a retrospective assessment of the clinical performance of the complete oral rehabilitation of patients with bruxism treated with implants and teeth-supported veneered and non-veneered monolithic zirconia restorations with increased occlusal vertical dimension. Methods: In this retrospective follow-up study, 16 bruxer patients, mean age 59.5 ± 14.9 years, were treated with 152 veneered and 229 non-veneered monolithic zirconia and followed for a mean of 58.8 ± 18.8 months (range 1–8 years). The patients were examined clinically and radiographically, annually. Clinical data were extracted from the medical records. In the recall appointments, modified California Dental Association (CDA) criteria were used to evaluate the restorations. Implant and restoration survival and success rates were recorded and analyzed. Results: The cumulative survival rates of implants and restorations were 97.7% and 97.6%, respectively. Nine restorations were replaced: three due to horizontal tooth fractures, two because of implant failure and four had secondary caries. A total of 43 biologic and technical complications were recorded. In the veneered group, the predominant complication was minor veneer chipping (16.4%), which required polishing only (grade 1). In the non-veneered group, the main complication was open proximal contacts between the implant restorations and adjacent teeth (14.5%). Conclusions: The survival rates of restorations and implants in patients with bruxism are excellent, even though veneered zirconia restoration exhibited a high rate of minor veneer chipping, which required polishing only. The biologic complication of fractured single-tooth abutment may occur.

## 1. Introduction

Bruxism, as defined by Lobbezoo et al. [1], is a repetitive jaw muscle activity characterized by clenching or grinding the teeth and/or by bracing or thrusting the mandible. This single definition was recently replaced with two separate definitions for sleeping and waking bruxism [2]. The prevalence of bruxism ranges from 8% to 31% and decreases with age [3]. Although patients with bruxism represent a significant proportion of the patient population, in most studies, bruxism is an exclusion criterion when evaluating survival and success of restorations since it may promote restoration fracture [4,5]. For this reason, information in the literature evaluating the restoration survival in bruxer patients is limited.

High-strength monolithic zirconia ceramic restorations have become the preferred treatment for patients with bruxism [6]. An overview of systematic reviews on zirconia restorations, reported satisfactory 5-year survival rates, with veneer fractures the predominant technical problem [7]. The author noted that in most of the studies, bruxism was excluded and was only discussed as a cause of possible technical complications.

In a recent study, comparing monolithic zirconia restorations in bruxer versus non-bruxer patients, no significant differences were found between the two groups regarding overall complications and survival rates [8]. However, in the anterior zone, whenever veneered feldspathic ceramic was added, a higher rate of chipping was noted in the bruxer group.

In a retrospective study on implant-supported, full-arch, fixed dental prostheses, with a mean follow-up of 10 years, Chrcanovic et al. [9] showed that bruxism was associated with implant and prosthesis failure, as well as with increased prevalence of technical complications.

Severe bruxism is often associated with extensive attrition and erosion that necessitates rehabilitation of complete dentition with an increased vertical dimension of occlusion (VDO). The question remains whether this change in VDO through extensive rehabilitation, especially in bruxer patients, causes increased rates of prosthetic complications.

In a multicenter retrospective clinical study, Fabbri et al. [10] found that functional and prosthetic complications with increased VDO in patients with full-arch tooth and implant rehabilitations were infrequent. Levartovsky et al. [11] evaluated the clinical performance of complete rehabilitation with zirconia restorations with increased VDO among bruxer patients. After a follow-up period of 28.2 ± 16.8 months, survival and success rates for all restorations were high. More studies with longer follow-up periods are needed to support these findings.

The aim of this retrospective study was to make retrospective assessment of the clinical performance of the complete oral rehabilitation of patients with bruxism treated with implants and teeth-supported veneered and non-veneered monolithic zirconia restorations with increased VDO.

## 2. Materials and Methods

### 2.1. Study Sample

Electronic records of patients with bruxism, who had been fully rehabilitated with monolithic zirconia restorations via an increased VDO, were screened from the records of the graduate clinic of prosthodontics in the Dental Medicine School and from the private practice of the program director (S.L.), who is an experienced prosthodontist.

### 2.2. Inclusion Criteria

Patients were included only if they were diagnosed with bruxism according to the international consensus of Lobbezoo et al. [1], underwent complete oral rehabilitation with an increased VDO using veneered and non-veneered monolithic zirconia restorations (Prettau, Zirkonzahn, Tel Aviv, Israel), had clinical and radiographic records and had a minimum follow-up of 1 year after final prosthesis.

### 2.3. Exclusion Criteria

The exclusion criteria were less than 1 year of follow-up, an uncontrolled medical condition, aggressive periodontal condition, incomplete records or unavailability for recall. The patients underwent clinical and radiographic examinations annually. These clinical data, as well as the preoperative data, were extracted from the medical records.

For each patient, the diagnostic grading system of “possible,” “probable” or “definite” sleeping or waking bruxism was recorded at the time of arrival, according to the international consensus [1] and the signs and symptoms of bruxism published by the International Classification of Sleep Disorders [12]. These patients were invited to attend a recall appointment to participate in the study and to report their usage of the occlusal splint during sleep.

### 2.4. Methods

**Figure 1:**
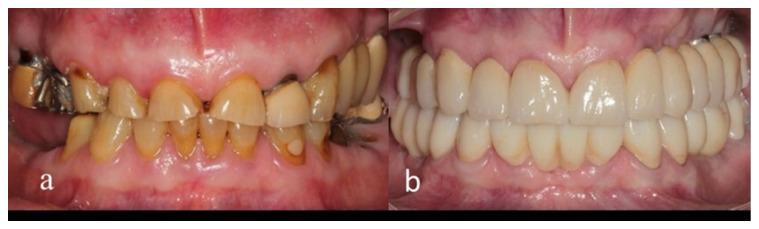
Patient 3: (**a**) Before treatment; (**b**) After treatment.

The detailed study protocol was previously described [11]. The same prosthetic approach was used for all patients, with an increase in VDO, which was determined according to the residual tooth structure and the space needed for the restorative materials (Figure 1a,b).

If the amount of the increased VDO was within the interocclusal rest space (IORS) of the patient, the temporary acrylic resin restorations were delivered immediately after mock-ups were tested. If the amount of the increased VDO was beyond the range of the IORS of the patient, a removable appliance was used for two months in order to test the adaptability of the new VDO. The temporary restorations were delivered only if no functional complications were reported [13].

After at least two months of normal function with the new VDO, final zirconia restorations were fabricated and delivered on teeth and implants, either as single abutments or short, fixed partial dentures with only one pontic. Implant-supported restorations were either screwed or cemented to the implant abutments.

The design of the non-veneered, monolithic zirconia was created using CAD-CAM software according to the manufacturer’s specifications (Prettau, Zirkonzahn, Tel Aviv, Israel). For the veneered monolithic zirconia design, facial cutback was carried out according to a virtual modification, and a feldspathic ceramic veneer was added to the nonfunctioning facial surfaces.

With the delivery of the final restorations, all patients received occlusal splints and were instructed to wear them during sleep.

### 2.5. Clinical and Radiographic Assessments and Classification of Observed Events at the Recall Appointment

Two examiners (S.L. and H.H.) were trained and independently assessed all radiographic and clinical records of the zirconia restorations and the abutments, implants and teeth. In case there was a disagreement in the rating of a certain restoration, the case was discussed by both examiners until they reached a consensus. Periapical radiographs of each implant and tooth abutment were taken using the long cone technique and were compared with the radiographs taken with the definitive prosthesis. Survival, biological and technical complications of all teeth, implants and prostheses were recorded.

For each implant, the following biological findings were recorded: periodontal probing depth, bleeding on probing, and peri-implant suppuration, if present. Implant survival was defined as a functioning implant with no clinical signs of infection, even if bone resorption was identified radiographically. Implant failure was defined as an implant that did not survive [14].

The monolithic zirconia restorations were assessed technically according to the modified California Dental Association (CDA) quality evaluation system for assessing surface, color, shape and marginal integrity [11,15,16]. Porcelain chipping was graded as described by Heintze and Rousson [17]: grade 1 = polishing, grade 2 = repair, grade 3 = replacement. A restoration that failed and had to be replaced or a tooth that had to be extracted was classified as absolute failure. Relative failure/complication was classified as any intervention (repair, polishing) by the dentist or by the laboratory due to compromised quality or impaired integrity (chipping, crack formation) [18].

### 2.6. Statistical Methods and Synthesis of Results

Descriptive statistics of patient age, follow-up time, and VDO increase were carried out using IBM SPSS statistics, version 25.0 (IBM Corp., Armonk, NY, USA). Life table analysis was used to assess implant and zirconia restoration survival. Kaplan–Meier curves were used to estimate the zirconia restoration survival and complication-free rates.

## 3. Results

The overall pool examined included 18 patients (6 females, 12 males) who met the inclusion criteria. Two did not attend the recall appointment; one did not respond to our invitations, and one was deceased. Therefore, only 16 patients (6 females, 10 males) attended the recall appointment. The participants ranged from 32 to 78 years of age (mean: 59.5 ± 14.9 years) and were followed for a mean observation period of 58.8 ± 18.8 months (range 1–8 years). All patients were diagnosed with “probable bruxism” [1]. They were treated with a total of 381 zirconia restorations and 88 implants via a VDO increase ranging from 2 to 7 mm (mean: 3.3 ± 1.6 mm), which was measured in the anterior region with calipers (Table 1).

No temporomandibular signs or symptoms were reported. Among the 88 implants restored, 19 (21.6%) were in the anterior region and 69 (78.4%) were in the posterior region of the jaws. Fifteen implants (17%) had cement-retained restorations, while the rest (83%) had screw-retained restorations.

The patients received restorations of a non-veneered monolithic zirconia design in the mandible and in the posterior quadrants of the maxilla. In the upper anterior crowns (incisors, canines and premolars), the buccal surfaces were veneered with a feldspathic ceramic material. Therefore, a total of 152 restorations were veneered monolithic zirconia, while the remaining 229 restorations were a non-veneered monolithic zirconia design. In the recall appointment, only seven patients reported using the occlusal splint during sleep (Table 1).

### 3.1. Biological Findings and Implant Survival Rate

Bleeding on probing was found on one or more teeth/implants in all patients. The periodontal probing depth was between 3 and 5 mm for all teeth and implants, except for two implants that failed during the follow-up period due to peri-implantitis. Thus, the cumulative implant survival rate was 97.7%. One implant, with a cement-retained restoration, failed in a female patient after 5 years (Lance Implant, MIS/Divident, Or Yehuda, Israel) and the other, with a screw-retained restoration, in another female patient, failed after 6 years (Tapered Screw-Vent^®^, Zimmer Biomet, Palm Beach Gardens, FL, USA). Both failed implants were placed as part of the rehabilitation and had no complication or inflammation around them before the rehabilitation. Life table survival analysis concerning implant survival rate is shown in Table 2.

### 3.2. Absolute Failure and Survival of Zirconia Restorations

The cumulative zirconia restoration survival rate was 97.6% due to nine failed restorations (Table 3).

**Figure 2:**
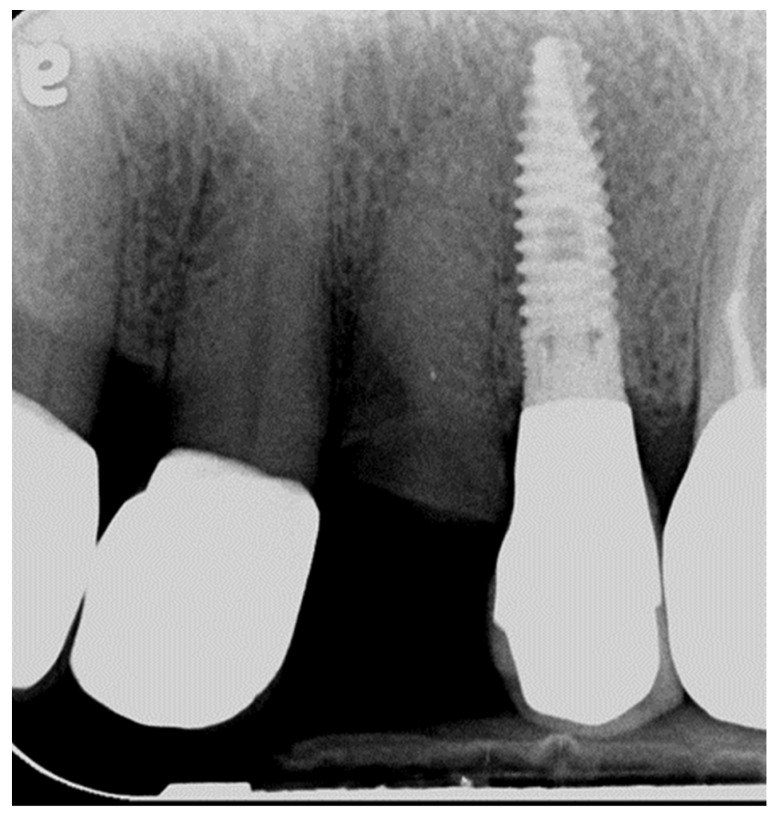
Patient 2—fractured first premolar.

Three teeth were fractured at the cemento-enamel junction (CEJ) in two patients. Patient 2 had two vital fractured premolars (first tooth after 1 year of function, the second after 4 years, Figure 2).

Patient 9 had a non-vital fractured canine after 5 years of function. Neither patient wore an occlusal splint (Table 4).

**Figure 3:**
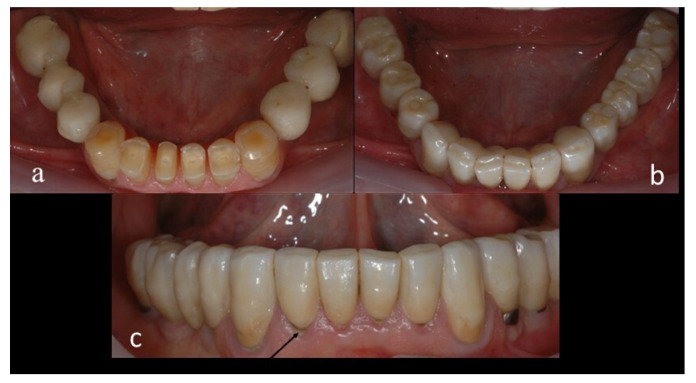
Patient 6: (**a**) before treatment; (**b**) after treatment; (**c**) secondary caries.

The 3 fractured teeth were replaced with implants and restored with new zirconia restorations. Four teeth (three in patient 6 and one in patient 8) had secondary caries, and their zirconia restorations had to be replaced (Figure 3a–c).

The last two failed restorations were of the failed implants. One was replaced with a new implant and a new zirconia restoration was delivered. The other implant was not replaced due to patient refusal.

### 3.3. Relative Failure and Success of the Treatments

The remaining failures were classified as complications (with/without repair). Among the 43 complications, 27 occurred in the veneered group and 16 in the non-veneered zirconia group (Table 5).

**Figure 4:**
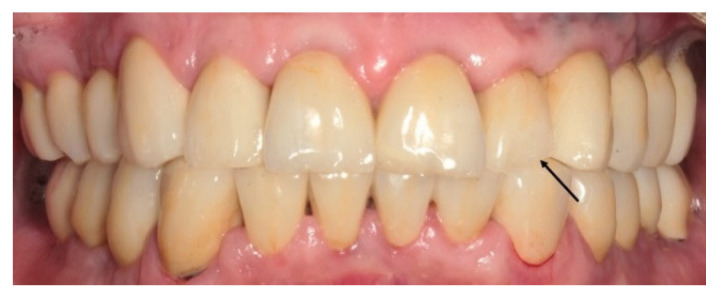
The arrow shows a chip-off of feldspathic porcelain in the incisal edge of a veneered monolithic zirconia restoration that needs only polishing.

Twenty-five feldspathic porcelain veneers in the anterior restorations (incisors and canines) chipped. All were on the incisal edge and required only polishing (grade 1) (Figure 4).

**Figure 5:**
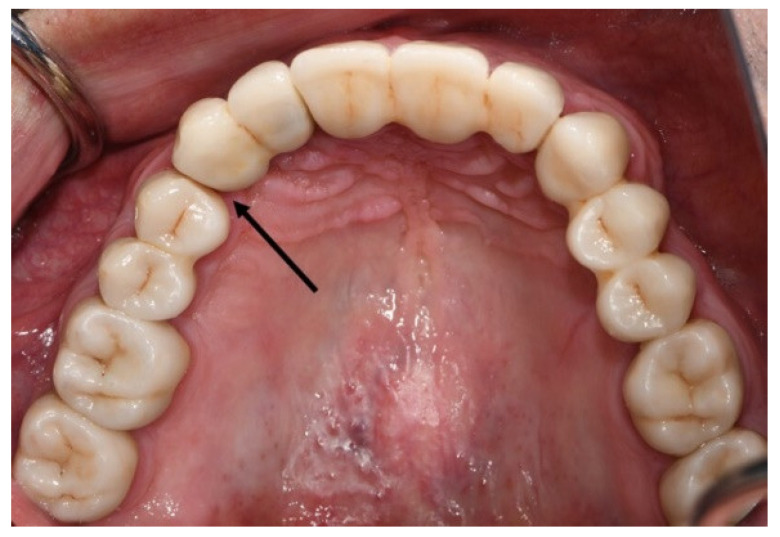
The arrow shows an open contact point between implant restoration and adjacent tooth that needs to be repaired because of impacted food.

Two minor zirconia fractures were observed in patient 16, which did not require any repair. Patient 7 experienced a retention loss of three cemented implant-supported restorations, and one loss of tooth vitality. The implant-supported restorations were recemented, and the non-vital tooth was treated with a root canal through an opening in the zirconia restoration. Twelve open contacts, affecting eight patients, were observed between implant restorations and the adjacent teeth: two were in the upper anterior region and the others in the posterior quadrants (Table 4). Eleven open contacts had no food impaction; therefore, no treatment was required, and the patients were carefully monitored. Only one open contact (upper premolar with a veneered monolithic zirconia) had impacted food (Figure 5).

Therefore, the implant restoration was removed, and feldspathic porcelain was added in order to achieve better contact with the adjacent tooth.

### 3.4. California Dental Association Ratings

The non-veneered and veneered monolithic zirconia restorations received separate CDA ratings.

**Figure 6:**
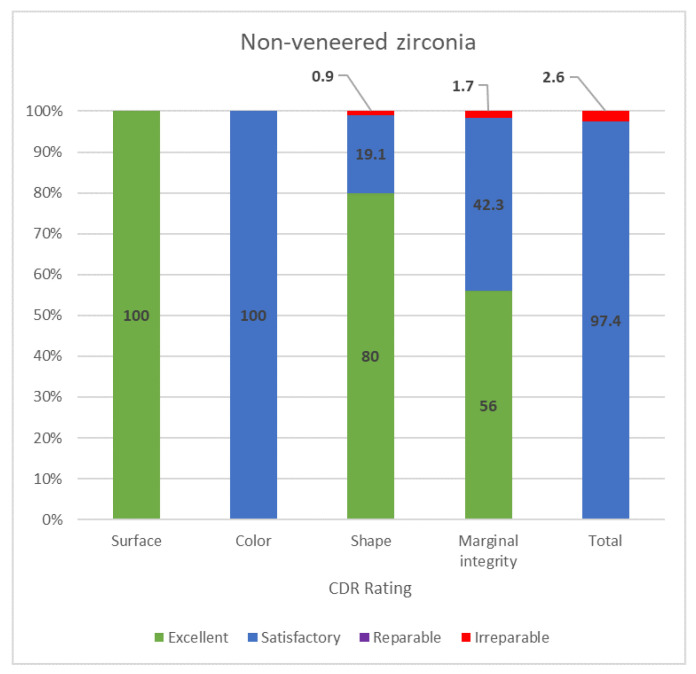
CDA ratings for the non-veneered zirconia group.

In the non-veneered monolithic zirconia restorations, the surfaces were excellent (100%) and the color was satisfactory (100%). The shape was excellent in 80% of the restorations, 0.9% were unsatisfactory-irreparable (two restorations were replaced due to implant failures) and 19.1% were satisfactory, including the 10 open contacts between implants and teeth without food impaction. The margins were excellent in 56% of the restorations; 1.7% were unsatisfactory and irreparable due to four secondary caries that needed to be replaced. The rest were rated as satisfactory with slight over-contouring (42.3%). Overall, 2.6% needed to be replaced, and the remaining 97.4% non-veneered monolithic zirconia restorations were evaluated as satisfactory and not in need of any repair or revision (Figure 6).

**Figure 7:**
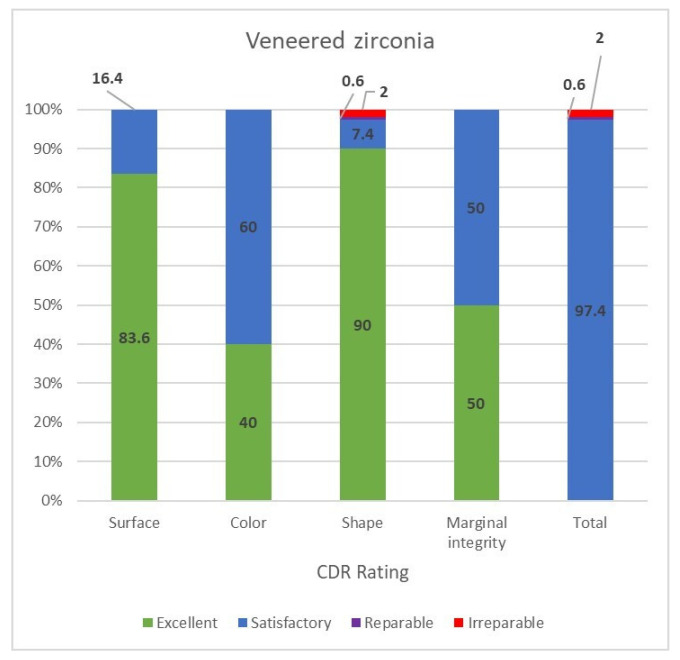
CDA ratings for the veneered zirconia group.

In the veneered monolithic zirconia restorations, the surfaces were excellent in 83.6% and the rest were satisfactory (16.4%) due to the minor veneer chipping, which required only polishing (grade 1). The color was rated as excellent in 40%, whereas 60% were rated as satisfactory. The shape was excellent in 90% of the restorations, 2.0% were unsatisfactory-irreparable (three restorations were replaced due to fractured teeth), 0.6% were unsatisfactory-reparable (one open contact that needed repair) and 7.4% were satisfactory in 7.4%, including the other open contact which did not have impacted food. Half of the restorations had excellent margins, while the rest were slightly over-contoured and rated as satisfactory. Overall, 2% needed to be replaced, 0.6% needed repair and the rest of the veneered monolithic zirconia restorations; 97.4% were evaluated as satisfactory (Figure 7).

**Figure 8:**
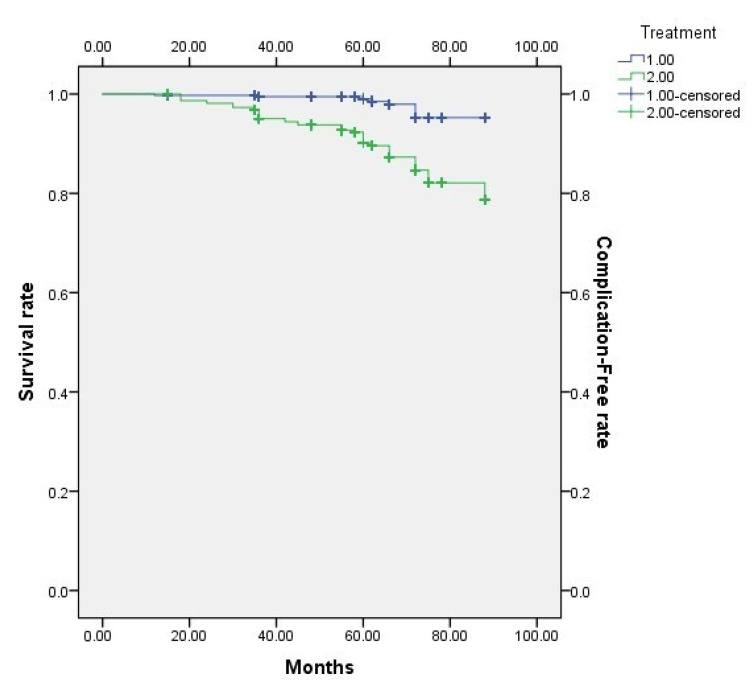
Kaplan–Meier overall survival rate combined with the complications that occurred.

The results of the Kaplan–Meier survival and complication-free rate analyses of all the zirconia restorations (veneered and non-veneered) are presented in Figure 8.

## 4. Discussion

The current study evaluated the clinical performance of the complete oral rehabilitation of patients with bruxism treated by implants and teeth-supported, veneered and non-veneered, monolithic zirconia restorations with an increased VDO after a follow-up of 1 to 8 years. The VDO was increased by 2 to 7 mm (mean 3.3 ± 1.6 mm). All patients adapted completely to the new VDO, without reporting any temporomandibular signs or symptoms throughout the follow-up time.

A total of 43 relative failures (complications) were divided between the two groups (Table 5). In the veneered monolithic zirconia group, a high complication rate of minor veneer chipping (16.4%) was observed. This finding agrees with previous studies where minor veneer chipping has been reported to occur more frequently in bruxers [8,19]. On the other hand, in the non-veneered zirconia group, only two minor zirconia fractures were observed. This is similar to other studies that showed that posterior monolithic zirconia restorations without porcelain veneer had high success rates even when bruxer patients were included [20,21].

In the current study, we used 3Y-TZP, which has high flexural strength (900–1200 MPa) and fracture toughness (9–10 MPa m^0.5^) but a major drawback of opacity [22]. Because of their high flexural strength, monolithic zirconia crowns have been widely accepted as a treatment of choice for heavy bruxers and patients with parafunctional habits, but because of their opacity, their use in the esthetic zone has been minimal. Therefore, in our study, we used labially veneered zirconia restorations in the anterior zone, on the buccal surfaces, to improve the esthetic appearance. A new version of zirconia with increased yttria content has been developed. It is fabricated with 5 mol% yttria that partially stabilizes the cubic phase and therefore, has improved optical properties. However, the more translucent the zirconia is, the lower its fracture strength [23]. Since the 5Y-TZP has significantly lower flexural strength than that of 3Y-TZP, it may not be suitable for bruxers, even in the anterior zone [24]. Recently, a new multi-layered translucent zirconia material, which is indicated for use in monolithic restorations in both the anterior and posterior regions, was introduced. The multi-layered zirconia has varying yttria contents in the different layers; thus, the strength and toughness of the layers with varying yttria contents are expected to differ [25]. In bruxers, the new monolithic multi-layered translucent zirconia may be used in the anterior zone; thus, eliminating the minor veneer chipping which was seen in our study.

Another technical complication, in the current study, was the open proximal contact between the implant restoration and the adjacent tooth, which occurred predominantly in the non-veneered zirconia group (14.5%). This is a common finding in other studies, regardless of bruxism. In a systematic review and meta-analysis, Bento et al. reported proximal contact loss rates of 41% with no significant difference between the posterior and anterior regions [26]. This result contrasts with ours, since 10 open contacts were found in the non-veneered group (mostly posterior) while only 2 were in the veneered group (mostly anterior region). This is due to the different number of implants placed in the anterior versus the posterior regions (19 vs. 69, respectively). In a review, Greenstein et al. showed that after an implant restoration is inserted in adjacent to a natural tooth, an interproximal gap developed 34% to 66% of the time [27]. Their recommendation was to wear an occlusal appliance during sleep in order to decrease the attrition of tooth contacts and reduce the open contacts, although they noted that this outcome has not been documented in the literature. Two years later, Zeng et al. [28] published a study that evaluated the effect of the vacuum-formed retainer on preventing proximal contact loss between the implant supported crown and its adjacent natural teeth. The 22 participants in the experimental group that wore a night guard, while the 24 participants in the control group received only examination. They found that the proximal contact loss rate on the mesial surface in the control group (62.5%) was significantly higher than in the experimental group (31.8%, χ^2^ = 4.330, *p* = 0.037) after a 1-year follow-up, but there was no statistical difference between the two groups on the distal surfaces. The open proximal contacts in the current study were found in eight patients, six of whom reported not wearing the night guard, while the other two were found in patients who reported using the splint (Table 4).

The cumulative zirconia restoration survival rate was 97.6% due to nine absolute failures (Table 3). An important biologic failure was noted in the veneered group in three teeth that fractured at the CEJ in two different patients (Table 5 and Figure 2). This is probably due to the high occlusal stress created by the bruxism. In addition, the stiff monolithic zirconia restoration, which is unable to absorb stresses, transmits the stress to the tooth. Eventually, the tooth fractures at its weakest point (below the covered surface of the crown). Since bruxism is an exclusion criterion in most studies, data on the survival and success of restorations and abutments in bruxer patients are lacking. Koenig et al. [29] included patients with bruxism while evaluating clinical outcomes of second-generation zirconia restorations. They reported that 80% of catastrophic failures and 76.9% of all complications occurred in the bruxer patients. To distribute the high occlusal forces in bruxer patients, when preparing teeth with minimal tooth structure, connecting two abutment teeth may be advised.

Other absolute failures were the secondary caries that occurred at the marginal integrity of four zirconia restorations and the two failed implants in the non-veneered group (Table 5 and Figure 6). A 1% incidence of secondary caries in all restorations occurred during the follow-up period, which agrees with other studies, according to the systematic review and meta-analysis of Leitão et al. [21] on clinical outcome of monolithic tooth-supported zirconia restorations. The low rate of implant failures observed in our study (cumulative survival rate of 97.7%, Table 2) might be explained by the small sample size of implants. These implants failed due to peri-implantitis and not due to implant disintegration, which may be present in bruxer patients who exert high occlusal stress. Because of the small number of implants, the difference between the survival of cemented or screw-retained implant restorations could not be analyzed.

Limitations of the current study include the retrospective design, in which clinical procedures are not standardized as they could be in a prospective follow-up. The small sample size and lack of a control group of non-bruxer patients can also be considered limitations of the present study. Additionally, the diagnosis of bruxism was based only on clinical inspection and self-report, without polysomnography examination. Additional, long-term, randomized, controlled trials with larger sample sizes are needed to assess the effect of bruxism on the survival of complete rehabilitation with monolithic zirconia restorations and increased VDO.

## 5. Conclusions

Within the limitations of this retrospective study, we conclude that complete oral rehabilitation using monolithic zirconia restorations with an increased VDO in patients with bruxism have high cumulative survival rates of implants and restorations (97.7% and 97.6%, respectively). In the veneered group, the predominant complication was minor veneer chipping (16.4%), which required only polishing (grade 1). In the non-veneered group, the major complication was open contacts between the implant restorations and the adjacent teeth (14.5%). A biologic complication of three fractured teeth, which were replaced with implant-supported restorations occurred in two patients. Therefore, in bruxer patients, when there is minimal tooth structure, connecting two adjacent abutment teeth may be advised.

## Figures and Tables

**Table 1:** Patient variables, restorations, materials and implants.

Patient Sex	Age at Treatment	VDO Increased (mm)	Follow-Up (Months)	NVZ Restorations	VZ Restorations	Total Restorations	Implants	Occlusal Splint
1 (F)	68	3	72	12	10	22	5	No
2 (M)	69	3	66	16	10	26	9	No
3 (M)	62	2	60	16	10	26	5	No
4 (M)	62	7	58	16	10	24	6	No
5 (F)	67	2	55	14	10	24	3	Yes
6 (M)	69	5	72	14	10	26	6	No
7 (M)	70	6	88	16	10	23	5	Yes
8 (F)	70	2	62	13	10	24	1	Yes
9 (M)	72	3	75	16	8	20	13	No
10 (F)	55	3	78	12	8	20	7	Yes
11 (M)	35	2	48	10	10	22	7	Yes
12 (M)	43	2	36	14	8	28	2	No
13 (F)	35	2	35	18	10	22	0	Yes
14 (M)	78	5	48	12	10	22	2	No
15 (M)	32	3	15	12	10	26	0	Yes
16 (F)	65	2	72	18	8	26	17	No
**Total**				229	152	381	88	

**Table 2:** Life table analysis of implant survival.

Interval (Years)	No. of Implants	No. of Failures	Interval Survival Rate (%)	Cumulative Survival Rate (%)
0–1	88	0	100	100
1–2	88	0	100	100
2–3	88	0	100	100
3–4	88	0	100	100
4–5	86	0	100	100
5–6	68	1	98.5	98.9
6–7	53	1	98.1	97.7
7–8	3	0	98.1	97.7

**Table 3:** Life table analysis of zirconia restoration survival.

Interval (Years)	No. of Zirconia Restorations	No. of Failures	Interval Survival Rate (%)	Cumulative Survival Rate (%)
0–1 year	381	1	99.7	99.7
1–2 years	381	0	99.7	99.7
2–3 years	359	1	99.7	99.5
3–4 years	331	0	99.7	99.5
4–5 years	309	1	99.7	99.2
5–6 years	217	2	99.0	98.7
6–7 years	142	4	97.2	97.6
7–8 years	26	0	97.2	97.6

**Table 4:** Patients and observed absolute and relative failures per patient.

Pt. # (Sex)	Fractured Tooth	Secondary Caries	Loss of Retention	Loss of Vitality	Porcelain Chipping	Zirconia Fracture (Minor)	Open Proximal Contact	Implant Failure	Occlusal Splint
1 (F)					3 *		1 *	1 #	No
2 (M)	2 #				5 *		2 **		No
3 (M)					2 *		1 *		No
4 (M)							2 *		No
5 (F)									Yes
6 (M)		3 *			3 *				Yes
7 (M)			3 **	1 **					Yes
8 (F)		1 *					1 **		Yes
9 (M)	1 *				3 *		2 *		No
10 (F)					2 *		1 *		Yes
11 (M)					3 *				Yes
12 (M)									No
13 (F)									Yes
14 (M)					2 *		2 *		No
15 (M)									Yes
16 (F)					2 *	2 *		1 *	No
**Total**	3	4	3	1	25	2	12	2	

# Absolute failure. * Relative failure with no repair. ** Relative failure with repair.

**Table 5:** Absolute (survival) and relative (complications) failures in the veneered and non-veneered zirconia groups.

Outcome	Zirconia Patient Group	
	Veneer	No Veneer
**Absolute failure (survival)**		
Secondary caries	0	4
Implant failure	0	2
Fractured tooth	3	0
**Total failures**	**3**	**6**
**Relative failure (complications)**		
Porcelain chipping	25	0
Zirconia fracture (minor)	0	2
Loss of retention	0	3
Loss of vitality	0	1
Open proximal contact	2	10
**Total complications**	27	16

## Data Availability

The data that support the findings of this study are available on request from the corresponding author. The data are not publicly available due to privacy or ethical restrictions.
